# Illuminating Intrinsically Disordered Proteins with Integrative Structural Biology

**DOI:** 10.3390/biom13010124

**Published:** 2023-01-07

**Authors:** Rachel Evans, Sravani Ramisetty, Prakash Kulkarni, Keith Weninger

**Affiliations:** 1 Department of Physics, North Carolina State University, Raleigh, NC 27695, USA; 2 Department of Medical Oncology and Therapeutics Research, City of Hope National Medical Center, Duarte, CA 91010, USA; 3 Department of Systems Biology, City of Hope National Medical Center, Duarte, CA 91010, USA

**Keywords:** intrinsically disordered proteins, integrative structural biology, unfolded, unstructured, flexible, protein function

## Abstract

Intense study of intrinsically disordered proteins (IDPs) did not begin in earnest until the late 1990s when a few groups, working independently, convinced the community that these ‘weird’ proteins could have important functions. Over the past two decades, it has become clear that IDPs play critical roles in a multitude of biological phenomena with prominent examples including coordination in signaling hubs, enabling gene regulation, and regulating ion channels, just to name a few. One contributing factor that delayed appreciation of IDP functional significance is the experimental difficulty in characterizing their dynamic conformations. The combined application of multiple methods, termed integrative structural biology, has emerged as an essential approach to understanding IDP phenomena. Here, we review some of the recent applications of the integrative structural biology philosophy to study IDPs.

## 1. Introduction

Intrinsically disordered proteins (IDPs) are presently a prime focus of the protein biochemistry research enterprise, but that was not always the case. Although IDPs represent around a third of all proteins in eukaryotes [1,2,3,4], they were not a fashionable topic for researchers until the late 1990s. The existence of natively unfolded or disordered segments within otherwise folded proteins was well known from the early days of X-ray crystallography where parts of proteins that were not part of structure solutions were presumed dynamic and flexible. Those regions were often excised to aid crystallization. A few examples of focused IDP study appear through the literature in the 1960s and 1970s [5]. The discrepancy of some protein mobilities in size-exclusion chromatography compared to well-folded standards was an early observation interpreted as being due to flexible, disordered proteins [6]. In the 1970s, NMR studies could reveal disordered conformations, for example in glucagon [7]. From the 1960s to the 1980s, components of ribosomes [5] and histones [8] were also considered to have flexibility or disorder.

Despite these studies discussing properties of IDPs, the idea that biological functions could derive directly from the disordered properties was generally not considered. Gradually, appreciation for functional impacts of flexible linkers between domains or disorder-to-order transitions accrued [9]. One notable example of ahead of its time thinking was Paul Sigler’s musings where he synthesized several results about transcription factors in 1988 [10], resulting in a proposal of a key functional role for the disordered domains. Perhaps, it was not more widely adopted in part following his naming of the functional domain as an ‘acid blob’ or ‘negative noodle’, alluding to the role of the overall charge of the disordered domain in this proposed function. The failure of the broader field to seriously consider that functions could directly result from the nature of the disordered chain in IDPs has been suggested by several authors [11,12,13] to be a result of the then dominance of the lock-and-key view of enzyme/substrate functional interactions, reinforced by the stunning successes of X-ray crystallography to provide snapshots of carefully folded proteins stable ‘active sites’. This bias, combined with the experimental difficulties in characterizing these disordered conformational ensembles, prevented earlier appreciation of the functional roles we now know IDPs have. Indeed, even today, IDPs are thought to comprise a significant fraction of the ‘dark’ proteome, the proteome that is genetically expected to exist but not yet observed and characterized [14].

In the 1990s, the proliferation of gene-based techniques to interrogate protein function, genomic sequencing, and bioinformatics advances, along with technical improvements in NMR, led to increased appreciation of the functional importance of IDPs. Uversky was among the first few scientists to discuss significant wide-spread functions for IDPs in ways that were highly influential and brought recognition of this potential to the wider field [15,16,17,18].

At the present time, we appreciate many important functions for IDPs in physiology. A few examples are coordinating signaling networks (hubs), contributing to gene regulation, and modulating ion channel function [19,20,21,22,23,24,25]. The mechanisms that underlie these functions are equally diverse: multi-valency, fuzzy complexes, hubs, switches, and non-genetic switches based on ensemble switching [25,26,27,28,29]. In addition to physiological functions, disease associated pathology involving IDPs are recognized with prominent examples including amyloid-β, Tau, and α-synuclein [30,31]. Recently, consideration of physio-chemical properties of IDPs, such as possible LLPS phenomena, have further expanded the sorts of functions that are contemplated for IDPs [32,33]. 

Some credit for establishing clear ‘structure-function’ paradigms arising from the disordered properties of IDPs must go to the practice of combining several distinct characterization approaches to draw conclusions, an approach termed integrative structural biology [34,35,36]. Integrative structural biology seeks to combine multiple characterization approaches with different sensitivities to provide a more complete understanding of biomolecular conformational ensembles and dynamics. The tendencies for IDPs to rapidly fluctuate while sampling wide ranges of conformation space rather than remaining in each state makes them well suited for applications of multiple experimental probes to reveal different aspects of their behaviors. Such integrative structural biology approaches are becoming more common. Impressively, Uversky anticipated the utility of multiple characterization methods to enhance the understanding of IDP functions. He amusingly illustrated the necessity of using integrative structural biology approaches for IDP studies with a parable about confusion when examining an elephant without the proper global perspective [37]. Here, we review some of the latest successes in combining methods through the integrative structural biology approach to characterize IDP conformations and address their myriad functions. 

## 2. Summary of Methods

**Figure 1:**
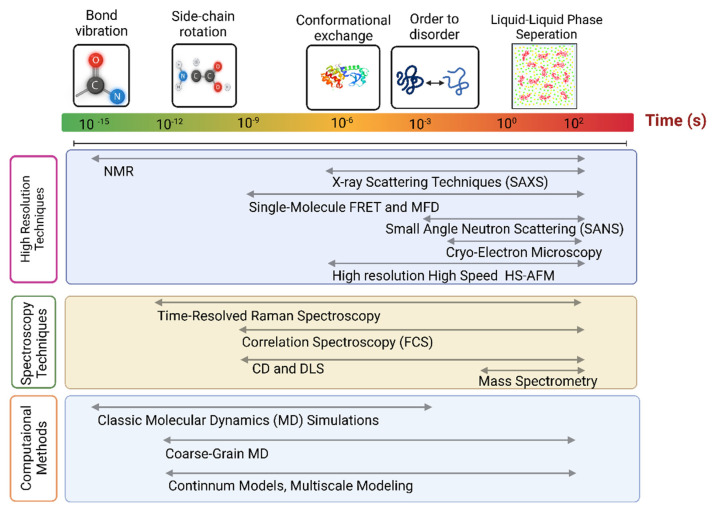
Comparison of sensitivities of methods commonly applied in IDP studies. Limits on temporal resolutions are not intended to be precise in this figure.

From a general experimental perspective, confirming that conclusions are consistent with multiple different experimental methods inspires increased confidence. For example, some methods require modification of molecules with extrinsic labels (fluorescence or EPR for example). Consistency with other measurement methods that do not use the modifications or use different modifications can confirm that such modifications do not affect the results in detrimental ways. In the integrative structural biology approach applied to IDPs, using different methods also has greater benefits because different methods have sensitivities to distinct length or time scales and even different concentration ranges (Figure 1). IDPs have behaviors that span broad ranges in these properties, making the use of multiple methods almost essential. For one example, liquid–liquid phase separation (LLPS) phenomena occur for a number of IDPs where concentrations are a key controlling factor [38]. Before discussing applications of integrative structural biology approaches to IDPs (see Figure 1), we first briefly describe some of the key individual methods used to characterize IDPs. 

### 2.1. Nuclear Magnetic Resonance

Nuclear magnetic resonance (NMR) has been used to study ordered proteins with atomic resolution since the 1950s [39,40] and applied to IDPs for several decades [37,41]. NMR relies on the local environment of each nucleus to produce a unique chemical shift signal which provides information on the conformation and close surroundings [37,42,43]. IDPs do not have a stable local environment so NMR alone lacks the ability to characterize disordered regions. However, NMR methods such as paramagnetic relaxation enhancement (PRE), secondary chemical shift (SCS), residual dipolar couplings (RDCs), and others can be used to characterize the conformational dynamics of IDP structures. Although NMR is a powerful technique, it is important to note that it is not without its limitations. Long IDPs must be divided into smaller sequences, experiments are often conducted at low temperatures which can decrease some kinetic activity, generally need high concentrations, and tags should be removed before conducting experiments [44]. NMR provides averages of ensembles but is limited in full conformational distribution determination for IDPs. A similar method, electron paramagnetic resonance (EPR), requires attachment of extrinsic spin labels and can be used at low temperatures to probe individual states and collect information on distance distributions [43,45].

### 2.2. Scattering Methods

Small angle X-ray scattering (SAXS) and small angle neutron scattering (SANS) are other methods commonly used to study IDPs which provide information on a global scale compared to the local scale NMR offers. A SAXS or SANS scattering profile can differentiate between globular and disordered proteins and determine a protein’s size and overall shape [46,47], although these interpretations are low-resolution, require high protein concentrations, and are dependent on model selection [48,49]. These methods are commonly used to characterize IDPs [47]. An IDP will react to changes in its environments that allow the protein to bind or unbind to other molecules present in the cell. By changing the experimental conditions to mimic these signals (pH, temperature, additives, etc.), the behavior of an IDP changes on a global scale, which SAXS and SNAS are well equipped to measure. 

### 2.3. Label-Based Approaches

Fluorescence correlation spectroscopy (FCS) is an optical technique commonly used to study the diffusion of fluorescently labeled molecular ensembles by measuring the time correlation of fluorescent fluctuations in the detected signal [50,51]. FCS is minimally invasive and does not require high protein concentrations [52,53]. As a solitary method, it is a powerful tool for studying the interactions between an IDP and its associated molecules, such as the Alzheimer’s related protein Tau and tubulin dimers [54]. Although FCS alone cannot reveal information about secondary protein structures, the conformational dynamics of a protein can be determined when it is combined with the results from other fluorescent methods [50]. Electron paramagnetic resonance (EPR) spectroscopy detects unpaired electrons and is commonly coupled with site-directed spin labeling (SDSL) to study a protein’s folding and unfolding events, interaction sites, and side chain mobility [45,55,56,57]. Although traditional labeling of a protein can change its conformational properties, the most common spin labels introduced via site-directed mutagenesis onto cysteine residues are relatively small, which decreases the risk deviating from wild-type behaviors [56,58]. SDSL-EPR spectroscopy is a sensitive and practical way to study the disorder-to-order transitions an IDP undergoes during binding events in near-native conditions [58,59]. This method is also capable of revealing IDPs or regions of an IDP that remain unstructured upon binding and complex formation [56]. Another EPR technique commonly used in the study of IDPs is Double Electron Electron Resonance (DEER), also called Pulsed Electron Double Resonance (PELDOR), which is well suited to determine spin site distances [45,60,61]. Because DEER requires spin-labeling, the distance measurements possess an inherent uncertainty due to potential (unintended) impacts on molecular conformation from the presence of the labels [45]. Multiparameter fluorescence detection (MFD) is an approach which collects fluorescent information such as intensity, lifetime, anisotropy, excitation and fluorescence spectra, and fluorescence quantum yield [62,63,64,65,66,67]. MFD is useful for improving the resolution of ensemble fluorescence experiments to reveal differences between similar sub-populations [65,67,68].

### 2.4. Single-Molecule Approaches

NMR, EPR (DEER) and SAXS are powerful methods that can be used to collect data about IDPs; however, the information provided is limited to the characteristics of an ensemble. Instead of averaging the properties of an ensemble, single molecule techniques can resolve dynamics and conformations of individual molecules [69,70,71]. Single molecule fluorescence (or Forster) resonance energy transfer (smFRET) is an optical spectroscopy approach to measuring the distance between two fluorophores of choice, but the fluorophore and position of labeling must be carefully considered to minimize the possibility of changing the dynamics of the protein. This is particularly useful in the study of IDPs because of the irregular folding dynamics of each protein as well as protein–protein interactions and protein aggregation [72,73,74,75]. smFRET has been applied to studies of many IDPs including the human proteins histone H1 and its partner nuclear protein prothymosin-alpha (ProTa), SNARE complexes such as syntaxin and SNAP-25, and Prostate-associated Gene 4 (PAGE4) [51,72,76,77,78,79,80,81,82,83,84,85,86,87]. 

### 2.5. Atomic Force Microscopy

Electron microscopy (EM) was among some of the first methods used to obtain structural data for proteins; however, it has had limited use for studying IDPs [34]. An exciting new technique being applied to study IDPs is high-speed AFM [88,89]. High-speed atomic force microscopy (HS-AFM) is a method particularly suited for studying protein functions in near-native conditions with no labeling necessary. HS-AFM has the capability to observe IDPs transitioning between states of order and disorder and partial folding under certain conditions with a broader range of applicable length scales than FRET [90]. Interactions with surfaces that might shift energy landscapes and thus conformational ensembles is a concern, but the practice of this method is advancing rapidly.

### 2.6. Cryo-EM and X-ray Crystallography

Cryo-electron microscopy (cryo-EM) is a rapidly advancing technique that is gaining popularity as a method for protein structural analysis [91]. Before the development of commercially available direct electron detectors and data analysis software for cryo-EM, X-ray crystallography was the method of choice to investigate protein structure [92]. However, X-ray crystallography does not provide insights on the properties of a disordered region due to its atomic flexibility, resulting in non-coherent X-rays. Instead, the lack of structural data, or missing electron density, is used to determine where disordered regions are located [37]. Unlike X-ray crystallography, cryo-EM does not require sample crystallization; instead, the proteins are frozen in a thin layer of solution [91,93,94]. Cryo-EM works well for proteins with large molecular weight and can survey multiple conformational states. However, similar to X-ray crystallography, cryo-EM only works with a moderate level of heterogeneity and regions of disorder are represented with poor resolution [92,93,95]. Time resolved measurements with both X-ray and cryo-EM methods for folded proteins are developing [96,97,98,99,100].

### 2.7. Solvent Accessibility Methods

Because solvent accessibility is associated with protein folding and stability, it can be a useful parameter when classifying and modeling an IDP [101]. 

#### 2.7.1. Hydrogen-Deuterium-Exchange

Hydrogen-deuterium-exchange (HDex or HDX) measures differences in deuterium uptake that are reflected in the solvent accessibility of the protein under native conditions in solution [102]. Information gathered from HDX is useful for studying folding intermediates as well as protein dynamics as the protein performs its function [102,103,104]. IDPs can be difficult to study using HDX because of their flexibility, heterogeneity in solution, and fast deuteration times [102]. Lowering the pH of the solution decreases the exchange rate and provides reasonable experimental time windows for the study of IDPs using HDX. To avoid the affect that lowering the pH can have on a protein’s structure and dynamics, pulse labeling HDX has been used to study IDPs [103,104,105]. 

#### 2.7.2. Crosslinking Mass Spectrometry

Crosslinking mass spectrometry (XL-MS) uses a “bottom-up” approach that supplies information on interaction sites rather than the “top-down” approach of native MS which informs overall protein structure [106]. XL-MS can be used to study the interaction sites between proteins or within a single protein. A cross-linking reaction, which can be performed in the protein’s native environment, covalently links nearby amino acids that react with the crosslinker of choice [104]. Another advantage of XL-MS is the low protein concentration required to perform experiments. Two residues often targeted in XL-MS are lysine and arginine which are frequently abundant in disordered regions or disordered proteins, causing XL-MS to gain popularity as a method for IDP studies [106,107,108,109]. However, studying dynamic proteins such as IDPs with XL-MS can be challenging because the results often reflect only a fraction of the conformations or residue distances of the ensemble [104]. 

#### 2.7.3. Proteolysis

**Figure 2:**
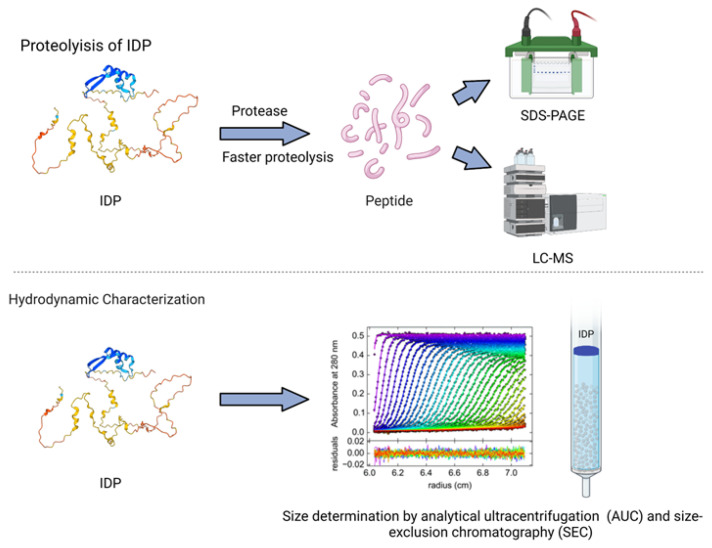
IDPs undergo faster proteolysis with enzymatic digestion compared to the structured proteins. The digested peptides are further analyzed using SDS-PAGE and LS-MS techniques. Techniques such as analytical ultracentrifugation and size-exclusion chromatography help to characterize the hydrodynamic size of IDPs.

Proteolysis, the enzymatic digestion of a protein into amino acids, disproportionally affects unstructured sequence regions [110]. IDPs are digested more quickly than ordered proteins due to their flexibility and the accessibility of protease susceptible sequences [13,111,112]. The rates of digestion are quantified via SDS-PAGE or liquid chromatography mass spectroscopy (Figure 2), which can then be used to loosely determine degree of disorder [113].

### 2.8. Spectroscopies

Spectroscopy is an invaluable tool for probing and studying characteristics of IDPs. 

#### 2.8.1. Circular Dichroism

Circular dichroism, the difference between the absorption coefficient of left- and right-handed circularly polarized light, is measured via circular dichroism (CD) spectroscopy [114]. CD spectroscopy is a powerful technique used to investigate secondary structure elements [115,116]. IDPs possess dynamic secondary structures that can be well assessed and characterized in an average sense by CD spectroscopy analysis [114,117]. Structural dynamics of an IDP can be reduced or promoted by altering their physical or chemical environment, which can then be quantified using CD spectroscopy. The two spectral regions used to study CD in proteins are the near-UV (250–300 nm) which correspond to the aromatic side chains and the far-UV (175–250 nm) that inform about secondary structures. Because an IDP moves through secondary structure as it changes conformations, far-UV CD spectroscopy is particularly useful for reporting the presence of alpha helices and beta sheets [118]. Time-resolved approaches using synchrotron light sources can provide information on dynamic processes in proteins down to nanosecond timescales [119], which eventually may prove useful for IDPs. 

#### 2.8.2. Fourier Transform Infrared Spectroscopy

Fourier transform infrared spectroscopy (FTIR) is another spectroscopic method used to study the secondary structure of proteins [120]. FTIR relies on the absorption of infrared light at the frequency of the sample’s molecular vibrational modes. The vibrational modes of a polypeptide chain, a repeated sequence of peptide bonds inherent to proteins, can produce up to nine bands measured by FTIR, the two most studied being the amide I and amide II bands [121]. Specifically, the amide I band is used to observe secondary structure formation. FTIR is also commonly used to study the aggregation of IDPs, such as the Parkinson’s disease associated IDP α-synuclein [120,122,123]. 

#### 2.8.3. Raman Spectroscopy

Raman spectroscopy obtains its name from its use of Raman scattering, or the inelastic scattering of light, due to a system’s molecular vibrations [121,124]. Comparable to FTIR spectroscopy, the measured change in energy from the incident light can be correlated to the protein’s vibrational modes and secondary structure [125,126]. Raman spectroscopy can be performed at dilute concentrations which is advantageous in the study of IDPs due to their aggregation tendences at high concentrations [127]. The conformational changes of an IDP are also well characterized by Raman spectral analysis. Raman optical activity (ROA) is another Raman scattering technique that measures the change in vibrational spectra due to left- and right-circularly polarized light and can add information about secondary and tertiary structures [124,128]. 

#### 2.8.4. Mass Spectrometry

Native mass spectrometry (MS) is a technique used in structural biology for studying the structure and stoichiometry of proteins through their mass to charge (m/z) ratio. MS has the capability to inform on multiple conformational states present in a heterogenous mixture and is often combined with other methods, such as ion mobility MS (IM-MS), which can separate the proteins by size and charge [104,129,130]. Time resolved MS has been successfully used to measure dynamic processes in proteins [131].

### 2.9. Hydrodynamic Characterizations

The hydrodynamic properties of a protein are necessary for conformation classification and can be determined with methods such as dynamic light scattering (DLS), FCS (see Section 2.3), size-exclusion chromatography (SEC, also known as gel filtration), and analytical ultracentrifugation (AUC) [46]. DLS, SEC, and AUC are complementary methods for studying the hydrodynamic (Stokes) radius, R_H_ [132]. DLS is a simple and noninvasive technique that can be used to obtain information on a protein’s hydrodynamic dimensions [133,134]. DLS measures the scattering of light caused by Brownian motion and has been applied to the study of IDPs with high aggregation tendencies [135,136]. SEC uses porous beads to separate molecules based on hydrodynamic dimensions [46]. AUC uses centrifugal force generated in a centrifuge to separate molecules based on their hydrodynamic properties (Figure 2). AUC can experimentally determine the sedimentation coefficient, s, which is inversely related to the Stokes radius [132]. Various combinations of these techniques, as well as molecular simulations, have been used to calculate and confirm the hydrodynamic characteristics of IDPs [78,137].

### 2.10. Computational Methods

All atom molecular dynamics simulation (MD simulation) is a computational method used to predict the behavior of proteins, especially when combined with parameters from data acquired via X-ray crystallography, SAXS, NMR, or other techniques [36]. MD has been increasingly applied to characterize conformational ensembles of IDPs [138,139,140,141,142]. MD simulation is a highly valuable tool for data analysis and structural modeling but is not without its limitations. Force fields that are used for MD simulations of structured proteins fail to succeed when applied to IDPs and the inhomogeneous conformational landscape occupied by any single IDP also presents modeling challenges [143]. MD simulations are a key tool in integrative structural biology due to their ability to combine information from many methods and create a unified model of a protein’s structure and conformational changes [144,145]. 

Until recently, a protein’s tertiary structure was unpredictable based on its amino acid sequence. The residue sequence in disordered regions varies in composition when compared to ordered proteins [146,147]. Several disordered regions of proteins have been predicted by a group of algorithms within the PONDR (predictor of natural disordered regions) family [148,149]. Using more than one predictor and averaging the results provide a more robust disorder profile than a single algorithm [144]. The associative memory, water-mediated, structure and energy model (AWSEM) is a course-grained force field model that has been used to predict protein structure, folding, and aggregation [144,150,151]. AWSEM’s optimized force fields have correctly predicted protein structures dependent solely on sequence [150,152,153]. AlphaFold, a machine learning model created by DeepMind, has made significant strides in the field of structural biology after successfully predicting the three-dimensional structure of proteins based on sequencing data [154]. Regions of low confidence in AlphaFold’s predictions correlate to disordered regions and confirm previous estimates that more than 30% of protein regions are disordered [154]. 

## 3. The Integrative Structural Biology Approach to IDPs and Examples

IDPs by nature fluctuate on many timescales among wide ranges of conformations. Their conformational ensembles can be altered by accessory proteins or post-translational modification. Thus, using an integrated, multiscale approach (integrative structural biology) rather than a single isolated technique is more prudent for accurately characterizing the dynamics and fluctuating conformational landscapes inherent to IDPs. Using a battery of methods with different sensitivities, complemented by advanced computational simulations, is essential to characterize the full range of the conformation space. Studying an IDP is like putting together a jigsaw puzzle where each method provides a limited number of pieces. Method one may gather all the blue pieces together, while method two helps arrange the edges. The full picture comes together only when the information from one method can be placed into its larger context with complementary methods. Therefore, instead of relying on the limited data provided by a single experimental method, integrative structural biology is an approach that combines the data from various methods to form models and a more complete understanding of these proteins [34,35,36,61,80,140,155,156,157,158,159,160,161,162,163]. 

As the application of integrative approaches to study IDPs is increasing at a rapid pace, here we will highlight only a few of the many successes. Our goal is to illustrate some different combinations of methods or cases where unexpected functions are uncovered. We will not discuss the important related topic of using combinations of methods to characterize dynamic assemblies of folded domains connected by disordered linkers [34,159,164,165,166].

### 3.1. Ubiquitin

To examine the robustness of an integrative structural biology approach, the ubiquitin protein in its denatured state was observed by combining results from multiple methods [156]. Ubiquitin is a regulatory protein involved in cell regulation with a tertiary structure that is denatured as urea concentration increases. Data collected from smFRET, NMR, and SAXS had good agreement for the distance distributions for unfolded ubiquitin. Local structure and dynamics were derived from NMR restraints while the overall shape was provided by SAXS measurements. Intramolecular distances and distributions within subpopulations as well as dynamic properties of the protein’s conformational changes were uncovered by smFRET. In this study, combining the results of smFRET, NMR, and SAXS provided a complete picture of the conformational ensemble of this unfolded protein. 

### 3.2. Nucleoporins

Phenylalanine-glycine-rich nucleoporins have also been studied using an integrative structural biology approach [167]. A combination of SAXS, smFRET measurements was compared with MD simulations that used different models for solvent interactions. The ultimate agreement of experiment and simulations in this work highlights successful approaches to improve theoretical force fields used to model IDPs. 

### 3.3. Aggregation-Prone Synaptic Proteins

The aggregation of some IDPs is associated with the pathology of diseases, such as α-Synuclein (αS) with Parkinson’s disease or amyloid-β (Aβ) and Tau with Alzheimer’s disease. αS has previously been investigated using X-ray diffraction [168] and NMR [157], but more recent studies [158] have used smFRET combined with MD simulations and NMR measurements to provide information on its structure and dynamics. Good agreement was found with other methods, and the conditions found to promote aggregation pointed toward possible therapeutic approaches to target αS.

Similarly, Aβ has been investigated [163]. Fluorophores were used to label both the N- and C- termini and FRET was observed in both free-diffusion and immobilized modes. Again, results aligned with previously reported data while adding information on possible reasons for aggregation of monomeric Aβ.

In the mid-1990s, before the wide acceptance of IDPs, studies of the Tau protein showed that its overall shape and conformation suggested it was similar to a denatured protein with no tertiary structure [169]. Since then, integrated structural biology has enhanced our understanding of these IDPs which are implicated in neurodegenerative disorders. There is evidence to support that both the aggregation of Tau and increased Tau-tubulin binding influence the pathology of disease. smFRET data combined with Monte Carlo simulations provide possible Tau conformations on binding to tubulin dimers [169].

### 3.4. Sic1

**Figure 3:**
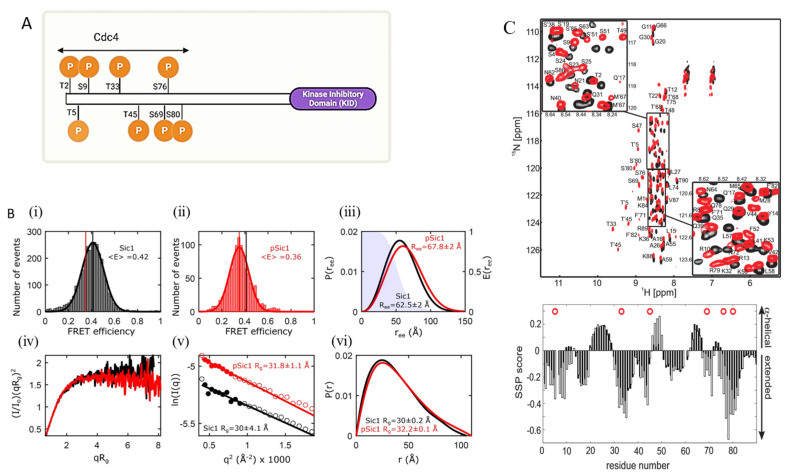
An integrative approach to elucidate IDP structure. (**A**) Schematic representation of full-length intrinsically disordered Sic1 protein and CPD phosphorylation sites in the N-terminal domain. The minimum functional KID fragment, the last 70 residues, is indicated in the purple box. (**B**) top three panels (**i**–**iii**) show smFRET efficiency histograms of Sic1 and pSic1 and end to end probability distributions. iv–vi show SAXS data for Sic1 and pSic1 and deduced *R_g_*, which was estimated to be approximately 30 Å for Sic1 and 32 Å for pSic1 [170]. (B) is adapted with permission from [170]. Copyright 2020, American Chemical Society. (**C**) Upper panel displays ^1^HN-^15^N correlation spectra of Sic1 (black) and pSic1 (red). The lower panel shows experimentally determined secondary structural propensity (SSP) values (described in [175]) for Sic1 (black bars) and pSic1 (open bars). Note that the helical vs. extended interpretations are marked on the right axis. Red circles indicate the locations of the phosphorylation sites [175]. (**C**) is reproduced with permission from [175]. Copyright 2008, National Academy of Sciences, USA.

In *Saccharomyces cerevisiae*, Sic1 is a disordered protein involved in cell cycle regulation and DNA replication initiation [170,171,172]. Sic1 forms a complex with a subunit of ubiquitin ligase, Cdc4, after the phosphorylation of at least six of the nine Cdc4 phosphodegron (CPD) sites on Sic1, seven of which are located on the 90 residue N-terminal (Figure 3A) [170,172]. Phosphorylation followed by ubiquitination results in the degradation of Sic1, which allows DNA replication to begin [170,171,173,174]. An integrative structural biology approach to Sic1 characterization that used NMR, SAXS, and smFRET (Figure 3B,C) focused on the seven CPD sites on the disordered N-terminal [170]. Phosphorylated Sic1 (pSic1) has different binding properties than Sic1, but neither phosphorylation nor Cdc4 binding creates a disorder-to-order transition of Sic1. SAXS and smFRET of both Sic1 and pSic1 were constrained by including NMR-PRE data and indicated a subtle conformational change in Sic1 after phosphorylation. Analysis of SAXS and smFRET data showed that these methods were individually capable of accurately measuring the root-mean-squared radius of gyration R_g_ and the root-mean-squared end-to-end distance R_e–e_, respectively. SAXS data alone show little change in conformational properties between Sic1 and pSic1; however, SAXS+PRE restrained ensembles show an expansion of R_e–e_ which is consistent with the change in distance observed by smFRET. 

### 3.5. N-WASP

An integrative approach allowed characterization of the conformational ensemble of the disordered domain of the neural Aldrich syndrome protein (N-WASP) [176], which regulates actin assembly pathways [177]. MD modeling generated conformational ensembles of the protein, which were validated by NMR and SAXS measurements. Using the SAXS and NMR data to benchmark simulations and guide selection of optimal force fields allowed the MD simulations to reveal both the global and local details of the conformational ensemble of this disordered protein. The simulations provided information about the transient underlying secondary structure within the ensembles. The use of experimentally derived restraints to guide computational modeling [178,179,180] or, more generally, cross validating simulation with experiments is a powerful tool to apply to IDP studies because it provides insights into both global and local structural features of the conformational ensembles.

### 3.6. SNAP-25

SNAP-25 is a SNARE protein that is a key player in neurotransmitter release. SNAP-25 is an intrinsically disordered protein that undergoes a disorder-to-order transition upon binding its partners syntaxin and synaptobrevin where it folds into colinear alpha helices to form the SNARE complex. SNARE complex formation is associated with membrane fusion of synaptic vesicles to the pre-synaptic terminal to release neurotransmitters. Integrated structural biology investigations of SNAP-25 combining smFRET, AUC, DLS, CD (circular dichroism) and SEC characterized the conformational ensemble in the isolated disordered state as consistent with a simple, semi-flexible polymer model with no underlying structure [78]. Interestingly, smFRET measurements of SNAP-25, in a binary complex with syntaxin (lacking synaptobrevin) that is on the pathway to full SNARE complex, found the transient tendency to switch between the folded alpha helix and a disordered conformation [87]. Returning to the isolated protein using additional methods of single molecule MFD, double electron-electron resonance (DEER), and MD, transient helix–coil transitions in short regions of SNAP-25 that occur in sub-millisecond timescales were observed despite a disordered fluctuating ensemble being the dominant conformational feature [161]. It was suggested that these transient alpha helix forming tendencies could play a role in priming SNAP-25 to zip into the SNARE complex rapidly upon binding with the requisite partners, assisting in the speed of neurotransmitter release. This example illustrates the value of the integrative structural biology approach for addressing measurements at the many length scales and timescales required to characterize IDP conformational ensembles, especially those with switching tendencies [82].

### 3.7. p27

p27 is a member of the Kip family of cyclin-dependent kinase inhibitors that plays an important role in controlling the cell cycle in eukaryotes [181]. Binding of the disordered C-terminal domain of p27 with cyclin-dependent kinase (Cdk2) and cyclins results in a disordered-to-ordered transition that has regulatory impact on the cell cycle. By integrating results from single-molecule multiparameter fluorescence spectroscopy, stopped-flow experiments, and molecular dynamics simulation, the multi-step process of assembling this fuzzy complex involving the disordered domain of p27 was mapped out [182,183].

The unbound p27 was found to be compact at the scale of a random coil by an integrated approach but rapidly fluctuating with dynamics covering orders of magnitudes of time scales from nanoseconds to milliseconds [184,185]. The interaction with its binding partners induced a multi-step process where p27 switches among conformational ensembles until favorable conformation is encountered to advance the binding process. In the end, p27 binds its partners in a more extended conformation than in isolation but remains dynamic without a fixed structure in a fuzzy complex. Elucidation of this pathway suggests that the assembly of the complex starts with a first recognition step involving conformational selections among rapidly fluctuating states, followed by a period waiting for a switch between distinct conformational sub-ensembles permitting progression to a later step where an induced fit phenomena completes the assembly. The complexity of the binding sequence is suggested to offer multiple opportunities for regulation of the assembly by other cellular signals.

### 3.8. PAGE4

Prostate-associated gene 4 (PAGE4) is an IDP that is expressed only in the prostate and only during early developmental stages and in the cancerous state [184]. An integrated structural biology approach identified phosphorylation-induced changes in the conformational ensemble of this IDP that were connected to impact cellular signaling pathway important to cancer progression. Combining experimental results from NMR, PRE, SAXS and smFRET studies with MD simulations revealed distinct changes in the conformational ensemble upon phosphorylation by different kinases [79,80,83,142,144,185,186]. In particular, phosphorylation by homeodomain-interacting protein kinase 1 (HIPK1) leads to a more compact ensemble, whereas phosphorylation by CDC-Like Kinase 2 (CLK2) expands the ensemble. The change in the conformational state was connected to signaling in prostate cancer by its ability to regulate interactions with the transcription factor c-Jun. HIPK1 treatment resulted in increased c-Jun dependent transcription activity in cell models of prostate cancer while CLK2 phosphorylation caused the opposite [79,80]. Given that CLK2 and PAGE4 are expressed only in androgen-dependent prostate cancer cells whereas HIPK1 is expressed in all prostate cancer cells (both androgen-dependent and -independent), these phosphorylation states that result in the expanded and contracted conformational ensembles were correlated with androgen sensitivity in prostate cancer [83,144,185,186,187,188,189]. Modeling these changing transcription factor interactions in a cellular androgen control pathway suggested that the PAGE4 phosphorylation state could oscillate in time, which could result in temporal oscillations of androgen sensitivity in prostate cancer [187,188,189]. This model suggests direct connections between changes in the conformational ensemble of an IDP and cell phenotypes in a cancer model. Such a complete picture would not have been obtained without the use of the integrated structural biology approach.

## 4. Summary and Conclusions

It took more than two decades for IDPs to be recognized as legitimate biological entities [190] with important functions in myriad biological functions from prebiotic evolution, multicellularity, and cell fate determination to phenotypic plasticity, adaptive evolution, and disease pathology. Several of Uversky’s contributions to the IDP field have shed new light on these important components of the proteome including remarkable conceptual advances from a dynamical systems perspective [191,192]. Therefore, this Special Issue of Biomolecules dedicated to VladimirUversky on his 60th birthday, is a celebration of his many contributions over the past three decades. 

## Data Availability

Not applicable.

## References

[1] Peng Z., Yan J., Fan X., Mizianty M.J., Xue B., Wang K., Hu G., Uversky V.N., Kurgan L. (2015). Exceptionally Abundant Exceptions: Comprehensive Characterization of Intrinsic Disorder in All Domains of Life. Cell. Mol. Life Sci..

[2] Xue B., Dunker A.K., Uversky V.N. (2012). Orderly Order in Protein Intrinsic Disorder Distribution: Disorder in 3500 Proteomes from Viruses and the Three Domains of Life. J. Biomol. Struct. Dyn..

[3] Ward J.J., Sodhi J.S., McGuffin L.J., Buxton B.F., Jones D.T. (2004). Prediction and Functional Analysis of Native Disorder in Proteins from the Three Kingdoms of Life. J. Mol. Biol..

[4] Dunker A.K., Obradovic Z., Romero P., Garner E.C., Brown C.J. (2000). Intrinsic Protein Disorder in Complete Genomes. Genome Inform. Ser. Workshop Genome Inform..

[5] DeForte S., Uversky V.N. (2016). Intrinsically Disordered Proteins in PubMed: What Can the Tip of the Iceberg Tell Us about What Lies Below?. RSC Adv..

[6] Chao L.-P., Roboz Einstein E. (1969). Estimation of the Molecular Weight of Flexible Disordered Proteins by Exclusion Chromatography. J. Chromatogr. A.

[7] Boesch C., Bundi A., Oppliger M., Wuthrich K. (1978). 1H Nuclear-Magnetic-Resonance Studies of the Molecular Conformation of Monomeric Glucagon in Aqueous Solution. Eur. J. Biochem..

[8] Boublik M., Bradbury E.M., Crane-Robinson C., Johns E.W. (1970). An Investigation of the Conformational Changes of Histone F2b by High Resolution Nuclear Magnetic Resonance. Eur. J. Biochem..

[9] Huber R., Bennett W.S. (1983). Functional Significance of Flexibility in Proteins. Biopolymers.

[10] Sigler P.B. (1988). Acid Blobs and Negative Noodles. Nature.

[11] Nishikawa K. (2009). Natively Unfolded Proteins: An Overview. Biophysics.

[12] Uversky V.N., Dunker A.K. (2010). Understanding Protein Non-Folding. Biochim. Biophys. Acta (BBA)—Proteins Proteom..

[13] Dyson H.J., Wright P.E. (2005). Intrinsically Unstructured Proteins and Their Functions. Nat. Rev. Mol. Cell Biol..

[14] Kulkarni P., Uversky V.N. (2018). Intrinsically Disordered Proteins: The Dark Horse of the Dark Proteome. Proteomics.

[15] Wright P.E., Dyson H.J. (1999). Intrinsically Unstructured Proteins: Re-Assessing the Protein Structure-Function Paradigm. J. Mol. Biol..

[16] Uversky V.N., Gillespie J.R., Fink A.L. (2000). Why Are “Natively Unfolded” Proteins Unstructured under Physiologic Conditions?. Proteins Struct. Funct. Genet..

[17] Tompa P. (2002). Intrinsically Unstructured Proteins. Trends Biochem. Sci..

[18] Dunker A.K., Lawson J.D., Brown C.J., Williams R.M., Romero P., Oh J.S., Oldfield C.J., Campen A.M., Ratliff C.M., Hipps K.W. (2001). Intrinsically Disordered Protein. J. Mol. Graph. Model..

[19] Ferrie J.J., Karr J.P., Tjian R., Darzacq X. (2022). “Structure”-Function Relationships in Eukaryotic Transcription Factors: The Role of Intrinsically Disordered Regions in Gene Regulation. Mol. Cell.

[20] Trnka M.J., Pellarin R., Robinson P.J. (2019). Role of Integrative Structural Biology in Understanding Transcriptional Initiation. Methods.

[21] Choi U.B., Kazi R., Stenzoski N., Wollmuth L.P., Uversky V.N., Bowen M.E. (2013). Modulating the Intrinsic Disorder in the Cytoplasmic Domain Alters the Biological Activity of the N-Methyl-D-Aspartatesensitive Glutamate Receptor. J. Biol. Chem..

[22] Hu G., Wu Z., Uversky V., Kurgan L. (2017). Functional Analysis of Human Hub Proteins and Their Interactors Involved in the Intrinsic Disorder-Enriched Interactions. Int. J. Mol. Sci..

[23] Deiana A., Forcelloni S., Porrello A., Giansanti A. (2019). Intrinsically Disordered Proteins and Structured Proteins with Intrinsically Disordered Regions Have Different Functional Roles in the Cell. PLoS ONE.

[24] Wright P.E., Dyson H.J. (2015). Intrinsically Disordered Proteins in Cellular Signalling and Regulation. Nat. Rev. Mol. Cell Biol..

[25] Kulkarni P., Leite V.B.P., Roy S., Bhattacharyya S., Mohanty A., Achuthan S., Singh D., Appadurai R., Rangarajan G., Weninger K. (2022). Intrinsically Disordered Proteins: Ensembles at the Limits of Anfinsen’s Dogma. Biophys. Rev..

[26] Dunker A.K., Cortese M.S., Romero P., Iakoucheva L.M., Uversky V.N. (2005). Flexible Nets. The Roles of Intrinsic Disorder in Protein Interaction Networks. FEBS J..

[27] Csermely P., Palotai R., Nussinov R. (2010). Induced Fit, Conformational Selection and Independent Dynamic Segments: An Extended View of Binding Events. Nat. Preced..

[28] Berlow R.B., Dyson H.J., Wright P.E. (2018). Expanding the Paradigm: Intrinsically Disordered Proteins and Allosteric Regulation. J. Mol. Biol..

[29] Fung H.Y.J., Birol M., Rhoades E. (2018). IDPs in Macromolecular Complexes: The Roles of Multivalent Interactions in Diverse Assemblies. Curr. Opin. Struct. Biol..

[30] Coskuner-Weber O., Mirzanli O., Uversky V.N. (2022). Intrinsically Disordered Proteins and Proteins with Intrinsically Disordered Regions in Neurodegenerative Diseases. Biophys. Rev..

[31] Martinelli A., Lopes F., John E., Carlini C., Ligabue-Braun R. (2019). Modulation of Disordered Proteins with a Focus on Neurodegenerative Diseases and Other Pathologies. Int. J. Mol. Sci..

[32] Dignon G.L., Best R.B., Mittal J. (2020). Biomolecular Phase Separation: From Molecular Driving Forces to Macroscopic Properties. Annu. Rev. Phys. Chem..

[33] Brangwynne C.P., Tompa P., Pappu R.v. (2015). Polymer Physics of Intracellular Phase Transitions. Nat. Phys..

[34] Rout M.P., Sali A. (2019). Principles for Integrative Structural Biology Studies. Cell.

[35] Ward A.B., Sali A., Wilson I.A. (2013). Integrative Structural Biology. Science.

[36] Masrati G., Landau M., Ben-Tal N., Lupas A., Kosloff M., Kosinski J. (2021). Integrative Structural Biology in the Era of Accurate Structure Prediction: The Era of Accurate Structure Prediction. J. Mol. Biol..

[37] Cohen I.R., Lajtha A., Lambris J.D., Paoletti R., Felli I.C., Pierattelli R. (2015). Intrinsically Disordered Proteins Studied by NMR Spectroscopy.

[38] Musacchio A. (2022). On the Role of Phase Separation in the Biogenesis of Membraneless Compartments. EMBO J..

[39] Saunders M., Wishnia A., Kirkwood J.G. (1957). The Nuclear Magnetic Resonance Spectrum of Ribonuclease. J. Am. Chem. Soc..

[40] Kowalsky A. (1962). Nuclear Magnetic Resonance Studies of Proteins. J. Biol. Chem..

[41] Dyson H.J., Wright P.E. (2021). NMR Illuminates Intrinsic Disorder. Curr. Opin. Struct. Biol..

[42] Mureddu L., Vuister G.W. (2019). Simple High-Resolution NMR Spectroscopy as a Tool in Molecular Biology. FEBS J..

[43] Konrat R. (2014). NMR Contributions to Structural Dynamics Studies of Intrinsically Disordered Proteins. J. Magn. Reson..

[44] Prestel A., Bugge K., Staby L., Hendus-Altenburger R., Kragelund B.B. (2018). Characterization of Dynamic IDP Complexes by NMR Spectroscopy.

[45] Drescher M. (2011). EPR in Protein Science.

[46] Fontana A., de Laureto P.P., Spolaore B., Frare E., Uversky V.N., Dunker A.K. (2012). Intrinsically Disordered Protein Analysis.

[47] Bernadó P., Svergun D.I. (2012). Structural Analysis of Intrinsically Disordered Proteins by Small-Angle X-ray Scattering. Mol. BioSyst..

[48] Pauw B.R. (2014). Corrigendum: Everything SAXS: Small-Angle Scattering Pattern Collection and Correction (2013 J. Phys.: Condens. Matter 25 383201). J. Phys. Condens. Matter.

[49] Fuertes G., Banterle N., Ruff K.M., Chowdhury A., Mercadante D., Koehler C., Kachala M., Estrada Girona G., Milles S., Mishra A. (2017). Decoupling of Size and Shape Fluctuations in Heteropolymeric Sequences Reconciles Discrepancies in SAXS vs. FRET Measurements. Proc. Natl. Acad. Sci. USA.

[50] Luitz M.P., Barth A., Crevenna A.H., Bomblies R., Lamb D.C., Zacharias M. (2017). Covalent Dye Attachment Influences the Dynamics and Conformational Properties of Flexible Peptides. PLoS ONE.

[51] Nasir I., Onuchic P.L., Labra S.R., Deniz A.A. (2019). Single-Molecule Fluorescence Studies of Intrinsically Disordered Proteins and Liquid Phase Separation. Biochim. Biophys. Acta (BBA)—Proteins Proteom..

[52] Yu L., Lei Y., Ma Y., Liu M., Zheng J., Dan D., Gao P. (2021). A Comprehensive Review of Fluorescence Correlation Spectroscopy. Front. Phys..

[53] Haustein E., Schwille P. (2007). Fluorescence Correlation Spectroscopy: Novel Variations of an Established Technique. Annu. Rev. Biophys. Biomol. Struct..

[54] Li X.-H., Culver J.A., Rhoades E. (2015). Tau Binds to Multiple Tubulin Dimers with Helical Structure. J. Am. Chem. Soc..

[55] Drescher M., Jeschke G. (2012). EPR Spectroscopy.

[56] Le Breton N., Martinho M., Mileo E., Etienne E., Gerbaud G., Guigliarelli B., Belle V. (2015). Exploring Intrinsically Disordered Proteins Using Site-Directed Spin Labeling Electron Paramagnetic Resonance Spectroscopy. Front. Mol. Biosci..

[57] Lorenzi M., Sylvi L., Gerbaud G., Mileo E., Halgand F., Walburger A., Vezin H., Belle V., Guigliarelli B., Magalon A. (2012). Conformational Selection Underlies Recognition of a Molybdoenzyme by Its Dedicated Chaperone. PLoS ONE.

[58] Klare J.P., Steinhoff H.-J. (2009). Spin Labeling EPR. Photosynth. Res..

[59] Longhi S., Belle V., Fournel A., Guigliarelli B., Carrière F. (2011). Probing Structural Transitions in Both Structured and Disordered Proteins Using Site-Directed Spin-Labeling EPR Spectroscopy. J. Pept. Sci..

[60] Van Doorslaer S., Murphy D.M. (2011). EPR Spectroscopy in Catalysis. EPR Spectroscopy.

[61] Peter M.F., Gebhardt C., Mächtel R., Muñoz G.G.M., Glaenzer J., Narducci A., Thomas G.H., Cordes T., Hagelueken G. (2022). Cross-Validation of Distance Measurements in Proteins by PELDOR/DEER and Single-Molecule FRET. Nat. Commun..

[62] Widengren J., Kudryavtsev V., Antonik M., Berger S., Gerken M., Seidel C.A.M. (2006). Single-Molecule Detection and Identification of Multiple Species by Multiparameter Fluorescence Detection. Anal. Chem..

[63] Ma J., Yanez-Orozco I.S., Rezaei Adariani S., Dolino D., Jayaraman V., Sanabria H. (2017). High Precision FRET at Single-Molecule Level for Biomolecule Structure Determination. J. Vis. Exp..

[64] Margittai M., Widengren J., Schweinberger E., Schröder G.F., Felekyan S., Haustein E., König M., Fasshauer D., Grubmüller H., Jahn R. (2003). Single-Molecule Fluorescence Resonance Energy Transfer Reveals a Dynamic Equilibrium between Closed and Open Conformations of Syntaxin 1. Proc. Natl. Acad. Sci. USA.

[65] Rothwell P.J., Berger S., Kensch O., Felekyan S., Antonik M., Wöhrl B.M., Restle T., Goody R.S., Seidel C.A.M. (2003). Multiparameter Single-Molecule Fluorescence Spectroscopy Reveals Heterogeneity of HIV-1 Reverse Transcriptase:Primer/Template Complexes. Proc. Natl. Acad. Sci. USA.

[66] Eggeling C., Berger S., Brand L., Fries J.R., Schaffer J., Volkmer A., Seidel C.A.M. (2001). Data Registration and Selective Single-Molecule Analysis Using Multi-Parameter Fluorescence Detection. J. Biotechnol..

[67] Hamilton G., Sanabria H. (2019). Multiparameter Fluorescence Spectroscopy of Single Molecules. Spectroscopy and Dynamics of Single Molecules.

[68] Sisamakis E., Valeri A., Kalinin S., Rothwell P.J., Seidel C.A.M. (2010). Accurate Single-Molecule FRET Studies Using Multiparameter Fluorescence Detection. Methods in Enzymology.

[69] LeBlanc S., Kulkarni P., Weninger K. (2018). Single Molecule FRET: A Powerful Tool to Study Intrinsically Disordered Proteins. Biomolecules.

[70] Holmstrom E.D., Holla A., Zheng W., Nettels D., Best R.B., Schuler B. (2018). Accurate Transfer Efficiencies, Distance Distributions, and Ensembles of Unfolded and Intrinsically Disordered Proteins from Single-Molecule FRET. Methods in Enzymology.

[71] Barth A., Opanasyuk O., Peulen T.O., Felekyan S., Kalinin S., Sanabria H., Seidel C.A.M. (2022). Unraveling Multi-State Molecular Dynamics in Single-Molecule FRET Experiments. I. Theory of FRET-Lines. J. Chem. Phys..

[72] Metskas L.A., Rhoades E. (2020). Single-Molecule FRET of Intrinsically Disordered Proteins. Annu. Rev. Phys. Chem..

[73] Hofmann H. (2016). Understanding Disordered and Unfolded Proteins Using Single-Molecule FRET and Polymer Theory. Methods Appl. Fluoresc..

[74] Schuler B., Soranno A., Hofmann H., Nettels D. (2016). Single-Molecule FRET Spectroscopy and the Polymer Physics of Unfolded and Intrinsically Disordered Proteins. Annu. Rev. Biophys..

[75] Tan P.S., Lemke E.A. (2018). Probing Differential Binding Mechanisms of Phenylalanine-Glycine-Rich Nucleoporins by Single-Molecule FRET. Methods Enzymol..

[76] Borgia A., Borgia M.B., Bugge K., Kissling V.M., Heidarsson P.O., Fernandes C.B., Sottini A., Soranno A., Buholzer K.J., Nettels D. (2018). Extreme Disorder in an Ultrahigh-Affinity Protein Complex. Nature.

[77] Sakon J.J., Weninger K.R. (2010). Detecting the Conformation of Individual Proteins in Live Cells. Nat. Methods.

[78] Choi U.B., McCann J.J., Weninger K.R., Bowen M.E. (2011). Beyond the Random Coil: Stochastic Conformational Switching in Intrinsically Disordered Proteins. Structure.

[79] Mooney S.M., Qiu R., Kim J.J., Sacho E.J., Rajagopalan K., Johng D., Shiraishi T., Kulkarni P., Weninger K.R. (2014). Cancer/Testis Antigen PAGE4, a Regulator of c-Jun Transactivation, Is Phosphorylated by Homeodomain-Interacting Protein Kinase 1, a Component of the Stress-Response Pathway. Biochemistry.

[80] He Y., Chen Y., Mooney S.M., Rajagopalan K., Bhargava A., Sacho E., Weninger K., Bryan P.N., Kulkarni P., Orban J. (2015). Phosphorylation-Induced Conformational Ensemble Switching in an Intrinsically Disordered Cancer/Testis Antigen. J. Biol. Chem..

[81] Gomes G.-N., Gradinaru C.C. (2017). Insights into the Conformations and Dynamics of Intrinsically Disordered Proteins Using Single-Molecule Fluorescence. Biochim. Biophys. Acta (BBA)—Proteins Proteom..

[82] Choi U.B., Sanabria H., Smirnova T., Bowen M.E., Weninger K.R. (2019). Spontaneous Switching among Conformational Ensembles in Intrinsically Disordered Proteins. Biomolecules.

[83] Rajagopalan K., Qiu R., Mooney S.M., Rao S., Shiraishi T., Sacho E., Huang H., Shapiro E., Weninger K.R., Kulkarni P. (2014). The Stress-Response Protein Prostate-Associated Gene 4, Interacts with c-Jun and Potentiates Its Transactivation. Biochim. Biophys. Acta (BBA)—Mol. Basis Dis..

[84] Hofmann H., Soranno A., Borgia A., Gast K., Nettels D., Schuler B. (2012). Polymer Scaling Laws of Unfolded and Intrinsically Disordered Proteins Quantified with Single-Molecule Spectroscopy. Proc. Natl. Acad. Sci. USA.

[85] Soranno A., Buchli B., Nettels D., Cheng R.R., Müller-Späth S., Pfeil S.H., Hoffmann A., Lipman E.A., Makarov D.E., Schuler B. (2012). Quantifying Internal Friction in Unfolded and Intrinsically Disordered Proteins with Single-Molecule Spectroscopy. Proc. Natl. Acad. Sci. USA.

[86] Brucale M., Schuler B., Samorì B. (2014). Single-Molecule Studies of Intrinsically Disordered Proteins. Chem. Rev..

[87] Weninger K., Bowen M.E., Choi U.B., Chu S., Brunger A.T. (2008). Accessory Proteins Stabilize the Acceptor Complex for Synaptobrevin, the 1:1 Syntaxin/SNAP-25 Complex. Structure.

[88] Miyagi A., Tsunaka Y., Uchihashi T., Mayanagi K., Hirose S., Morikawa K., Ando T. (2008). Visualization of Intrinsically Disordered Regions of Proteins by High-Speed Atomic Force Microscopy. ChemPhysChem.

[89] Kodera N., Ando T. (2022). Guide to Studying Intrinsically Disordered Proteins by High-Speed Atomic Force Microscopy. Methods.

[90] Kodera N., Ando T. (2022). Visualization of Intrinsically Disordered Proteins by High-Speed Atomic Force Microscopy. Curr. Opin. Struct. Biol..

[91] Nwanochie E., Uversky V.N. (2019). Structure Determination by Single-Particle Cryo-Electron Microscopy: Only the Sky (and Intrinsic Disorder) Is the Limit. Int. J. Mol. Sci..

[92] Benjin X., Ling L. (2020). Developments, Applications, and Prospects of Cryo-electron Microscopy. Protein Sci..

[93] Abriata L.A., Dal Peraro M. (2020). Will Cryo-Electron Microscopy Shift the Current Paradigm in Protein Structure Prediction?. J. Chem. Inf. Model..

[94] Musselman C.A., Kutateladze T.G. (2021). Characterization of Functional Disordered Regions within Chromatin-Associated Proteins. iScience.

[95] Bonomi M., Vendruscolo M. (2019). Determination of Protein Structural Ensembles Using Cryo-Electron Microscopy. Curr. Opin. Struct. Biol..

[96] Schmidt M. (2021). Macromolecular Movies, Storybooks Written by Nature. Biophys. Rev..

[97] Brändén G., Neutze R. (2021). Advances and Challenges in Time-Resolved Macromolecular Crystallography. Science.

[98] Malla T.N., Schmidt M. (2022). Transient State Measurements on Proteins by Time-Resolved Crystallography. Curr. Opin. Struct. Biol..

[99] Frank J. (2017). Time-Resolved Cryo-Electron Microscopy: Recent Progress. J. Struct. Biol..

[100] Dandey V.P., Budell W.C., Wei H., Bobe D., Maruthi K., Kopylov M., Eng E.T., Kahn P.A., Hinshaw J.E., Kundu N. (2020). Time-Resolved Cryo-EM Using Spotiton. Nat. Methods.

[101] Ali S., Hassan M., Islam A., Ahmad F. (2014). A Review of Methods Available to Estimate Solvent-Accessible Surface Areas of Soluble Proteins in the Folded and Unfolded States. Curr. Protein Pept. Sci..

[102] Hodge E.A., Benhaim M.A., Lee K.K. (2020). Bridging Protein Structure, Dynamics, and Function Using Hydrogen/Deuterium-exchange Mass Spectrometry. Protein Sci..

[103] Zhang Y., Rempel D.L., Zhang J., Sharma A.K., Mirica L.M., Gross M.L. (2013). Pulsed Hydrogen–Deuterium Exchange Mass Spectrometry Probes Conformational Changes in Amyloid Beta (Aβ) Peptide Aggregation. Proc. Natl. Acad. Sci. USA.

[104] Beveridge R., Calabrese A.N. (2021). Structural Proteomics Methods to Interrogate the Conformations and Dynamics of Intrinsically Disordered Proteins. Front. Chem..

[105] Illes-Toth E., Rempel D.L., Gross M.L. (2018). Pulsed Hydrogen–Deuterium Exchange Illuminates the Aggregation Kinetics of α-Synuclein, the Causative Agent for Parkinson’s Disease. ACS Chem. Neurosci..

[106] Piersimoni L., Kastritis P.L., Arlt C., Sinz A. (2022). Cross-Linking Mass Spectrometry for Investigating Protein Conformations and Protein–Protein Interactions─A Method for All Seasons. Chem. Rev..

[107] Ubbiali D., Fratini M., Piersimoni L., Ihling C.H., Kipping M., Heilmann I., Iacobucci C., Sinz A. (2022). Direct Observation of “Elongated” Conformational States in A-Synuclein upon Liquid-Liquid Phase Separation. Angew. Chem. Int. Ed..

[108] Chen D., Drombosky K.W., Hou Z., Sari L., Kashmer O.M., Ryder B.D., Perez V.A., Woodard D.R., Lin M.M., Diamond M.I. (2019). Tau Local Structure Shields an Amyloid-Forming Motif and Controls Aggregation Propensity. Nat. Commun.

[109] Niemeyer M., Moreno Castillo E., Ihling C.H., Iacobucci C., Wilde V., Hellmuth A., Hoehenwarter W., Samodelov S.L., Zurbriggen M.D., Kastritis P.L. (2020). Flexibility of Intrinsically Disordered Degrons in AUX/IAA Proteins Reinforces Auxin Co-Receptor Assemblies. Nat. Commun.

[110] Suskiewicz M.J., Sussman J.L., Silman I., Shaul Y. (2011). Context-Dependent Resistance to Proteolysis of Intrinsically Disordered Proteins. Protein Sci..

[111] Johnson D.E., Xue B., Sickmeier M.D., Meng J., Cortese M.S., Oldfield C.J., le Gall T., Dunker A.K., Uversky V.N. (2012). High-Throughput Characterization of Intrinsic Disorder in Proteins from the Protein Structure Initiative. J. Struct. Biol..

[112] Baker E.S., Luckner S.R., Krause K.L., Lambden P.R., Clarke I.N., Ward V.K. (2012). Inherent Structural Disorder and Dimerisation of Murine Norovirus NS1-2 Protein. PLoS ONE.

[113] Hamdi K., Salladini E., O’Brien D.P., Brier S., Chenal A., Yacoubi I., Longhi S. (2017). Structural Disorder and Induced Folding within Two Cereal, ABA Stress and Ripening (ASR) Proteins. Sci. Rep..

[114] Chemes L.B., Alonso L.G., Noval M.G., de Prat-Gay G. (2012). Circular Dichroism Techniques for the Analysis of Intrinsically Disordered Proteins and Domains. Intrinsically Disordered Protein Analysis.

[115] Micsonai A., Moussong É., Murvai N., Tantos Á., Tőke O., Réfrégiers M., Wien F., Kardos J. (2022). Disordered–Ordered Protein Binary Classification by Circular Dichroism Spectroscopy. Front. Mol. Biosci..

[116] Ezerski J.C., Zhang P., Jennings N.C., Waxham M.N., Cheung M.S. (2020). Molecular Dynamics Ensemble Refinement of Intrinsically Disordered Peptides According to Deconvoluted Spectra from Circular Dichroism. Biophys. J..

[117] Uversky V.N. (2002). Natively Unfolded Proteins: A Point Where Biology Waits for Physics. Protein Sci..

[118] Na J.-H., Lee W.-K., Yu Y. (2018). How Do We Study the Dynamic Structure of Unstructured Proteins: A Case Study on Nopp140 as an Example of a Large, Intrinsically Disordered Protein. Int. J. Mol. Sci..

[119] Auvray F., Dennetiere D., Giuliani A., Jamme F., Wien F., Nay B., Zirah S., Polack F., Menneglier C., Lagarde B. (2019). Time Resolved Transient Circular Dichroism Spectroscopy Using Synchrotron Natural Polarization. Struct. Dyn..

[120] Natalello A., Ami D., Doglia S.M. (2012). Fourier transform infrared spectroscopy of intrinsically disordered proteins: Measurement procedures and data analyses. Intrinsically Disordered Protein Analysis.

[121] Uversky V.N. (2015). Biophysical Methods to Investigate Intrinsically Disordered Proteins: Avoiding an “Elephant and Blind Men” Situation. Adv. Exp. Med. Biol..

[122] Sethi A., Anunciado D., Tian J., Vu D.M., Gnanakaran S. (2013). Deducing Conformational Variability of Intrinsically Disordered Proteins from Infrared Spectroscopy with Bayesian Statistics. Chem. Phys..

[123] Takekiyo T., Yamada N., Nakazawa C.T., Amo T., Asano A., Yoshimura Y. (2020). Formation of A-synuclein Aggregates in Aqueous Ethylammonium Nitrate Solutions. Biopolymers.

[124] Zhu F., Isaacs N.W., Hecht L., Barron L.D. (2005). Raman Optical Activity: A Tool for Protein Structure Analysis. Structure.

[125] Sane S.U., Cramer S.M., Przybycien T.M. (1999). A Holistic Approach to Protein Secondary Structure Characterization Using Amide I Band Raman Spectroscopy. Anal. Biochem..

[126] Berjot M., Marx J., Alix A.J.P. (1987). Determination of the Secondary Structure of Proteins from the Raman Amide I Band: The Reference Intensity Profiles Method. J. Raman Spectrosc..

[127] Maiti N.C., Apetri M.M., Zagorski M.G., Carey P.R., Anderson V.E. (2004). Raman Spectroscopic Characterization of Secondary Structure in Natively Unfolded Proteins: α-Synuclein. J. Am. Chem. Soc..

[128] Syme C.D., Blanch E.W., Holt C., Jakes R., Goedert M., Hecht L., Barron L.D. (2002). A Raman Optical Activity Study of Rheomorphism in Caseins, Synucleins and Tau: New Insight into the Structure and Behaviour of Natively Unfolded Proteins. Eur. J. Biochem..

[129] Stuchfield D., France A.P., Migas L.G., Thalhammer A., Bremer A., Bellina B., Barran P.E. (2018). The Use of Mass Spectrometry to Examine IDPs: Unique Insights and Caveats.

[130] Santambrogio C., Natalello A., Brocca S., Ponzini E., Grandori R. (2019). Conformational Characterization and Classification of Intrinsically Disordered Proteins by Native Mass Spectrometry and Charge-State Distribution Analysis. Proteomics.

[131] Lento C., Wilson D.J. (2022). Subsecond Time-Resolved Mass Spectrometry in Dynamic Structural Biology. Chem. Rev..

[132] Salvay A.G., Communie G., Ebel C. (2012). Sedimentation Velocity Analytical Ultracentrifugation for Intrinsically Disordered Proteins.

[133] Gast K., Fiedler C. (2012). Dynamic and Static Light Scattering of Intrinsically Disordered Proteins. Intrinsically Disordered Protein Analysis.

[134] Al-Ghobashy M.A., Mostafa M.M., Abed H.S., Fathalla F.A., Salem M.Y. (2017). Correlation between Dynamic Light Scattering and Size Exclusion High Performance Liquid Chromatography for Monitoring the Effect of PH on Stability of Biopharmaceuticals. J. Chromatogr. B.

[135] Leite J.P., Gimeno A., Taboada P., Jiménez-Barbero J.J., Gales L. (2020). Dissection of the Key Steps of Amyloid-β Peptide 1–40 Fibrillogenesis. Int. J. Biol. Macromol..

[136] Hochmair J., Exner C., Betzel C., Mandelkow E., Wegmann S. (2023). Light Microscopy and Dynamic Light Scattering to Study Liquid-Liquid Phase Separation of Tau Proteins In Vitro. Protein Aggregation.

[137] Tomasso M.E., Tarver M.J., Devarajan D., Whitten S.T. (2016). Hydrodynamic Radii of Intrinsically Disordered Proteins Determined from Experimental Polyproline II Propensities. PLoS Comput. Biol..

[138] Wang W. (2021). Recent Advances in Atomic Molecular Dynamics Simulation of Intrinsically Disordered Proteins. Phys. Chem. Chem. Phys..

[139] Dokholyan N.V. (2020). Experimentally-Driven Protein Structure Modeling. J. Proteom..

[140] Hsu C.C., Buehler M.J., Tarakanova A. (2020). The Order-Disorder Continuum: Linking Predictions of Protein Structure and Disorder through Molecular Simulation. Sci. Rep..

[141] Best R.B. (2017). Computational and Theoretical Advances in Studies of Intrinsically Disordered Proteins. Curr. Opin. Struct. Biol..

[142] Lin X., Roy S., Jolly M.K., Bocci F., Schafer N.P., Tsai M.-Y., Chen Y., He Y., Grishaev A., Weninger K. (2018). PAGE4 and Conformational Switching: Insights from Molecular Dynamics Simulations and Implications for Prostate Cancer. J. Mol. Biol..

[143] Kasahara K., Terazawa H., Takahashi T., Higo J. (2019). Studies on Molecular Dynamics of Intrinsically Disordered Proteins and Their Fuzzy Complexes: A Mini-Review. Comput. Struct. Biotechnol. J..

[144] Lin X., Kulkarni P., Bocci F., Schafer N.P., Roy S., Tsai M.Y., He Y., Chen Y., Rajagopalan K., Mooney S.M. (2019). Structural and Dynamical Order of a Disordered Protein: Molecular Insights into Conformational Switching of Page4 at the Systems Level. Biomolecules.

[145] Blackledge M., Ferrage F., Kadeřávek P., Salvi N., Zapletal V., Jaseňáková Z., Zachrdla M., Padrta P., Narasimhan S., Marquardsen T. (2022). Convergent Views on Disordered Protein Dynamics from NMR and Computational Approaches. Biophys. J..

[146] Romero P., Obradovic Z., Li X., Garner E.C., Brown C.J., Dunker A.K. (2001). Sequence Complexity of Disordered Protein. Proteins Struct. Funct. Genet..

[147] Obradovic Z., Peng K., Vucetic S., Radivojac P., Dunker A.K. (2005). Exploiting Heterogeneous Sequence Properties Improves Prediction of Protein Disorder. Proteins Struct. Funct. Bioinform..

[148] Xue B., Dunbrack R.L., Williams R.W., Dunker A.K., Uversky V.N. (2010). PONDR-FIT: A Meta-Predictor of Intrinsically Disordered Amino Acids. Biochim. Biophys. Acta (BBA)—Proteins Proteom..

[149] Peng K., Radivojac P., Vucetic S., Dunker A.K., Obradovic Z. (2006). Length-Dependent Prediction of Protein Intrinsic Disorder. BMC Bioinform..

[150] Sirovetz B.J., Schafer N.P., Wolynes P.G. (2017). Protein Structure Prediction: Making AWSEM AWSEM-ER by Adding Evolutionary Restraints. Proteins Struct. Funct. Bioinform..

[151] Kmiecik S., Gront D., Kolinski M., Wieteska L., Dawid A.E., Kolinski A. (2016). Coarse-Grained Protein Models and Their Applications. Chem. Rev..

[152] Chen M., Lin X., Zheng W., Onuchic J.N., Wolynes P.G. (2016). Protein Folding and Structure Prediction from the Ground Up: The Atomistic Associative Memory, Water Mediated, Structure and Energy Model. J. Phys. Chem. B.

[153] Chen M., Lin X., Lu W., Onuchic J.N., Wolynes P.G. (2017). Protein Folding and Structure Prediction from the Ground Up II: AAWSEM for α/β Proteins. J. Phys. Chem. B.

[154] Ruff K.M., Pappu R.v. (2021). AlphaFold and Implications for Intrinsically Disordered Proteins. J. Mol. Biol..

[155] Ehm T., Shinar H., Meir S., Sekhon A., Sethi V., Morgan I.L., Rahamim G., Saleh O.A., Beck R. (2021). Intrinsically Disordered Proteins at the Nano-Scale. Nano Futures.

[156] Aznauryan M., Delgado L., Soranno A., Nettels D., Huang J., Labhardt A.M., Grzesiek S., Schuler B. (2016). Comprehensive Structural and Dynamical View of an Unfolded Protein from the Combination of Single-Molecule FRET, NMR, and SAXS. Proc. Natl. Acad. Sci. USA.

[157] Dedmon M.M., Lindorff-Larsen K., Christodoulou J., Vendruscolo M., Dobson C.M. (2005). Mapping Long-Range Interactions in α-Synuclein Using Spin-Label NMR and Ensemble Molecular Dynamics Simulations. J. Am. Chem. Soc..

[158] Ferrie J.J., Haney C.M., Yoon J., Pan B., Lin Y.-C., Fakhraai Z., Rhoades E., Nath A., Petersson E.J. (2018). Using a FRET Library with Multiple Probe Pairs To Drive Monte Carlo Simulations of α-Synuclein. Biophys. J..

[159] Hamilton G.L., Saikia N., Basak S., Welcome F.S., Wu F., Kubiak J., Zhang C., Hao Y., Seidel C.A., Ding F. (2022). Fuzzy Supertertiary Interactions within PSD-95 Enable Ligand Binding. eLife.

[160] Thomasen F.E., Lindorff-Larsen K. (2022). Conformational Ensembles of Intrinsically Disordered Proteins and Flexible Multidomain Proteins. Biochem. Soc. Trans..

[161] Saikia N., Yanez-Orozco I.S., Qiu R., Hao P., Milikisiyants S., Ou E., Hamilton G.L., Weninger K.R., Smirnova T.I., Sanabria H. (2021). Integrative Structural Dynamics Probing of the Conformational Heterogeneity in Synaptosomal-Associated Protein 25. Cell Rep. Phys. Sci..

[162] Choi U.B., Xiao S., Wollmuth L.P., Bowen M.E. (2011). Effect of Src Kinase Phosphorylation on Disordered C-Terminal Domain of N-Methyl-D-Aspartic Acid (NMDA) Receptor Subunit GluN2B Protein. J. Biol. Chem..

[163] Meng F., Bellaiche M.M.J., Kim J.-Y., Zerze G.H., Best R.B., Chung H.S. (2018). Highly Disordered Amyloid-β Monomer Probed by Single-Molecule FRET and MD Simulation. Biophys. J..

[164] Brunger A.T., Strop P., Vrljic M., Chu S., Weninger K.R. (2011). Three-Dimensional Molecular Modeling with Single Molecule FRET. J. Struct. Biol..

[165] Choi U.B., Strop P., Vrljic M., Chu S., Brunger A.T., Weninger K.R. (2010). Single-Molecule FRET–Derived Model of the Synaptotagmin 1–SNARE Fusion Complex. Nat. Struct. Mol. Biol..

[166] Lerner E., Barth A., Hendrix J., Ambrose B., Birkedal V., Blanchard S.C., Börner R., Sung Chung H., Cordes T., Craggs T.D. (2021). FRET-Based Dynamic Structural Biology: Challenges, Perspectives and an Appeal for Open-Science Practices. eLife.

[167] Mercadante D., Milles S., Fuertes G., Svergun D.I., Lemke E.A., Gräter F. (2015). Kirkwood–Buff Approach Rescues Overcollapse of a Disordered Protein in Canonical Protein Force Fields. J. Phys. Chem. B.

[168] Araki K., Yagi N., Nakatani R., Sekiguchi H., So M., Yagi H., Ohta N., Nagai Y., Goto Y., Mochizuki H. (2016). A Small-Angle X-ray Scattering Study of Alpha-Synuclein from Human Red Blood Cells. Sci. Rep..

[169] Schweers O., Schönbrunn-Hanebeck E., Marx A., Mandelkow E. (1994). Structural Studies of Tau Protein and Alzheimer Paired Helical Filaments Show No Evidence for Beta-Structure. J. Biol. Chem..

[170] Gomes G.-N.W., Krzeminski M., Namini A., Martin E.W., Mittag T., Head-Gordon T., Forman-Kay J.D., Gradinaru C.C. (2020). Conformational Ensembles of an Intrinsically Disordered Protein Consistent with NMR, SAXS, and Single-Molecule FRET. J. Am. Chem. Soc..

[171] Liu B., Chia D., Csizmok V., Farber P., Forman-Kay J.D., Gradinaru C.C. (2014). The Effect of Intrachain Electrostatic Repulsion on Conformational Disorder and Dynamics of the Sic1 Protein. J. Phys. Chem. B.

[172] Gomes G.-N.W., Namini A., Gradinaru C.C. (2022). Integrative Conformational Ensembles of Sic1 Using Different Initial Pools and Optimization Methods. Front. Mol. Biosci..

[173] Sala D., Cosentino U., Ranaudo A., Greco C., Moro G. (2020). Dynamical Behavior and Conformational Selection Mechanism of the Intrinsically Disordered Sic1 Kinase-Inhibitor Domain. Life.

[174] Nash P., Tang X., Orlicky S., Chen Q., Gertler F.B., Mendenhall M.D., Sicheri F., Pawson T., Tyers M. (2001). Multisite Phosphorylation of a CDK Inhibitor Sets a Threshold for the Onset of DNA Replication. Nature.

[175] Mittag T., Orlicky S., Choy W., Tang X., Lin H., Sicheri F., Kay L.E., Tyers M., Forman-Kay J.D. (2008). Dynamic equilibrium engagement of a polyvalent ligand with a single-site receptor. Proc. Natl. Acad. Sci. USA.

[176] Chan-Yao-Chong M., Deville C., Pinet L., van Heijenoort C., Durand D., Ha-Duong T. (2019). Structural Characterization of N-WASP Domain V Using MD Simulations with NMR and SAXS Data. Biophys. J..

[177] Hansen M.D.H., Kwiatkowski A.V. (2013). Control of Actin Dynamics by Allosteric Regulation of Actin Binding Proteins. Int. Rev. Cell Mol. Biol..

[178] Robustelli P., Piana S., Shaw D.E. (2018). Developing a Molecular Dynamics Force Field for Both Folded and Disordered Protein States. Proc. Natl. Acad. Sci. USA.

[179] Chan-Yao-Chong M., Durand D., Ha-Duong T. (2019). Molecular Dynamics Simulations Combined with Nuclear Magnetic Resonance and/or Small-Angle X-ray Scattering Data for Characterizing Intrinsically Disordered Protein Conformational Ensembles. J. Chem. Inf. Model..

[180] Rauscher S., Gapsys V., Gajda M.J., Zweckstetter M., de Groot B.L., Grubmüller H. (2015). Structural Ensembles of Intrinsically Disordered Proteins Depend Strongly on Force Field: A Comparison to Experiment. J. Chem. Theory Comput..

[181] Ou L., Waddell M.B., Kriwacki R.W. (2012). Mechanism of Cell Cycle Entry Mediated by the Intrinsically Disordered Protein P27 Kip1. ACS Chem. Biol..

[182] Tsytlonok M., Hemmen K., Hamilton G., Kolimi N., Felekyan S., Seidel C.A.M., Tompa P., Sanabria H. (2020). Specific Conformational Dynamics and Expansion Underpin a Multi-Step Mechanism for Specific Binding of P27 with Cdk2/Cyclin A. J. Mol. Biol..

[183] Tsytlonok M., Sanabria H., Wang Y., Felekyan S., Hemmen K., Phillips A.H., Yun M.-K., Waddell M.B., Park C.-G., Vaithiyalingam S. (2019). Dynamic Anticipation by Cdk2/Cyclin A-Bound P27 Mediates Signal Integration in Cell Cycle Regulation. Nat. Commun..

[184] Das R.K., Huang Y., Phillips A.H., Kriwacki R.W., Pappu R.v. (2016). Cryptic Sequence Features within the Disordered Protein P27 Kip1 Regulate Cell Cycle Signaling. Proc. Natl. Acad. Sci. USA.

[185] Zeng Y., He Y., Yang F., Mooney S.M., Getzenberg R.H., Orban J., Kulkarni P. (2011). The Cancer/Testis Antigen Prostate-Associated Gene 4 (PAGE4) Is a Highly Intrinsically Disordered Protein. J. Biol. Chem..

[186] Kulkarni P., Jolly M.K., Jia D., Mooney S.M., Bhargava A., Kagohara L.T., Chen Y., Hao P., He Y., Veltri R.W. (2017). Phosphorylation-Induced Conformational Dynamics in an Intrinsically Disordered Protein and Potential Role in Phenotypic Heterogeneity. Proc. Natl. Acad. Sci. USA.

[187] Kulkarni P., Dunker A., Weninger K., Orban J. (2016). Prostate-Associated Gene 4 (PAGE4), an Intrinsically Disordered Cancer/Testis Antigen, Is a Novel Therapeutic Target for Prostate Cancer. Asian J. Androl..

[188] Salgia R., Jolly M., Dorff T., Lau C., Weninger K., Orban J., Kulkarni P. (2018). Prostate-Associated Gene 4 (PAGE4): Leveraging the Conformational Dynamics of a Dancing Protein Cloud as a Therapeutic Target. J. Clin. Med..

[189] Jolly M.K., Kulkarni P., Weninger K., Orban J., Levine H. (2018). Phenotypic Plasticity, Bet-Hedging, and Androgen Independence in Prostate Cancer: Role of Non-Genetic Heterogeneity. Front. Oncol..

[190] Uversky V.N., Kulkarni P. (2021). Intrinsically Disordered Proteins: Chronology of a Discovery. Biophys. Chem..

[191] Uversky V.N. (2013). Unusual Biophysics of Intrinsically Disordered Proteins. Biochim. Biophys. Acta.

[192] Kulkarni P., Bhattacharya S., Achuthan S., Behal A., Jolly M.K., Kotnala S., Mohanty A., Rangarajan G., Salgia R., Uversky V. (2022). Intrinsically Disordered Proteins: Critical Components of the Wetware. Chem. Rev..

